# Serum γ-glutamyltransferase level and incidence risk of metabolic syndrome in community dwelling adults: longitudinal findings over 12 years

**DOI:** 10.1186/s13098-023-01000-5

**Published:** 2023-02-23

**Authors:** Jiwon Kwak, In-Ho Seo, Yong-Jae Lee

**Affiliations:** Department of Family Medicine, Yonsei University College of Medicine, Gangnam Severance Hospital, 211 Eonju-ro, Gangnam-Gu, Seoul, 06273 Republic of Korea

**Keywords:** Metabolic syndrome, γ-glutamyltransferase, Oxidative stress, Epidemiology

## Abstract

**Purpose:**

Although a recent meta-analysis demonstrated a positive association between serum γ-glutamyltransferase (GGT) and metabolic syndrome (MetS), sex differences in the relationship between GGT levels and MetS risk were not fully considered. We prospectively examined the relationship between serum GGT levels and incidence risk of MetS.

**Methods:**

Data were collected from the Korean Genome and Epidemiology Study (KoGES) enrolled in 2001–2002. Among 10,030 total participants, 5960 adults (3130 men and 2830 women) aged 40–69 without MetS were included and divided according to sex-specific quartiles of baseline serum GGT levels and followed up biennially until 2014. The hazard ratios (HRs) with 95% confidence intervals (CIs) for incident MetS were prospectively analyzed using multiple Cox proportional hazards regression analysis models.

**Results:**

Among 5960 participants, 1215 males (38.8%) and 1263 females (44.6%) developed MetS during 12-year follow up. Higher quartiles of GGT showed significantly higher cumulative incidence of MetS in both sexes (log-rank test P < 0.001). The HRs (95% CIs) for incident type 2 diabetes for the highest quartile versus referent lowest quartile for serum GGT levels were 3.01 (2.35–3.76) for men and 1.83 (1.30–2.57) for women after adjusting for age, smoking status, daily alcohol intake (g/day), regular exercise, family history of diabetes, and log-transformed LDL-cholesterol, creatinine, and aminotransferase levels.

**Conclusion:**

In conclusion, high levels of GGT were found to be associated with increased risk of Mets in both men and women and the positive associations were stronger in men than in women.

## Introduction

Metabolic syndrome (MetS) is a cluster of cardiometabolic risk factors including visceral obesity, high blood pressure, hyperglycemia, and atherogenic dyslipidemia. Individuals with MetS are more susceptible to type 2 diabetes, cardiovascular disease (CVD), and several cancers, which are major leading causes of death [1, 2]. In addition, the prevalence of MetS has increased in recent decades, up to approximately 30% worldwide, and this increase is most prevalent in developed countries [3]. Given the economic and health burden of MetS, early identification of individuals at higher risk for developing MetS is an important concern from a public health perspective [4]. Despite the fact that the pathophysiology of MetS is not clearly elucidated, accumulating evidence suggests that insulin resistance and low-grade inflammation accompanied by oxidative stress play a key role in the development of MetS [5].

The γ-glutamyltransferase (GGT) is a microsomal membrane binding protein present in serum and on the external surface of most cells, particularly in the liver and kidney [6, 7]. GGT plays an important role in glutathione homeostasis, which has both lipophilic and hydrophilic antioxidant capacities against free radicals and oxidative stress [8, 9]. Although an elevated GGT level is traditionally regarded as a serologic marker of alcohol consumption or hepatobiliary disease [7], epidemiological studies suggest that higher GGT levels are an independent predictor for CVD morbidity and mortality [10]. Moreover, a recent longitudinal research study showed that GGT is useful in predicting subsequent development of incident type 2 diabetes among community dwelling Korean adults [11]. A previous meta-analysis on serum GGT levels and incidence risk of MetS showed a positive association between serum GGT levels and MetS in nine prospective cohort studies [12]. However, the sex-specific relationship between GGT levels and the risk of MetS has not been fully explored in previous studies. Limited research suggests that there may be sex differences in the effect of GGT on the development of MetS, with small sample sizes or limited follow-up duration. Furthermore, previous research has shown that the relationship between GGT and cardiovascular risk factors, such as hypertension and diabetes mellitus, can vary between sexes [13, 14]. Additionally, GGT levels, which are closely linked to alcohol consumption, have different distributions in men and women, and the definition of MetS is often applied differently between sexes by various organizations.

In this regard, sex-specific analysis of GGT’s long term impact on MetS is required to provide an accurate understanding of the association between GGT levels and MetS risk. Therefore, we prospectively examined the relationship between serum GGT levels and the incident MetS risk using sex-specific analysis from a large community-based Korean cohort observed over 12 years in middle-aged and older men and women.

## Methods

### Study population

This study was derived from the Korean Genome and Epidemiology Study (KoGES), a large prospective cohort study initiated by the Korea National Institute of Health to investigate the prevalence of and risk factors for chronic diseases in Korea (KCDC; http://www.cdc.go.kr/CDC/eng/main.jsp). Details of the KoGES and the sampling method have been reported previously [15]. The KoGES comprises six prospective cohort studies including community-based prospective cohort studies such as the (1) KoGES-Ansan and Ansung, (2) KoGES-health examinee (HEXA), and (3) KoGES-cardiovascular disease association study (CAVAS) study, and gene-environment model studies such as (4) KoGES-twin and family, (5) KoGES-immigrant, and (6) KoGES-emigrant (Japan and China) study. We used secondary dataset from the community -based KoGES- Ansan and Ansung study.

**Fig. 1:**
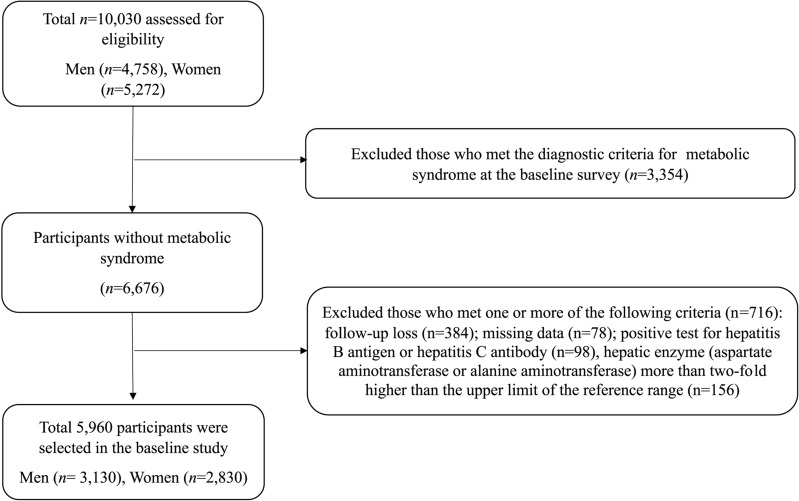
Flow chart for selection of the study population

Recruited participants were community dwelling, aged over 40, and living in Ansan (an urban area) or Ansung (a rural area) for at least 6 months at the time of enrollment. Participants were enrolled in 2001–2002 and followed-up biennially until 2014. Among 10,030 participants (4758 men and 5272 women) assessed at the baseline survey, we excluded 3354 (33.4%) participants who had met the diagnostic criteria for MetS. Of the remaining participants (n = 6676), we also excluded those who met one or more of the following criteria (n = 716): follow-up loss (n = 384); missing data (n = 78); positive test for hepatitis B antigen or hepatitis C antibody (n = 98), hepatic enzyme (aspartate aminotransferase or alanine aminotransferase) more than two-fold higher than the upper limit of the reference range (n = 156). After these exclusions, 5960 participants (3130 men and 2830 women) were selected during the baseline survey (Fig. 1). This study was approved by the Ethics Committee of the Korean Health and Genomic Study at the Korea National Institute of Health. All patients voluntarily enrolled and provided written informed consent. This study was conducted in accordance with the Declaration of Helsinki.

### Anthropometric and clinical measurements

Waist circumference, body weight, and height were measured according to a standard protocol. All researchers followed these instructions to measure waist circumference: locate the top of the hip bone (iliac crest) and take the measurement just above this bony landmark, where one finger can fit between the iliac crest and the lowest rib. Blood pressure was measured after the subject had rested for 5 min in a sitting position (Baumanometer, Baum Co. Inc., N.Y.). Systolic blood pressure and diastolic blood pressure were defined as the average of both arm readings. Smoking status, drinking behavior, physical activity, medication use and family history of diabetes mellitus data were also collected with self-reported questionnaires. Smoking status among participants was divided into two categories: current smokers and non-smokers. The status of alcohol consumption was categorized as current drinking (at more than twice per week) or not drinking and daily alcohol intake was expressed as g/day [16]. Physical activity was classified into two categories according to the frequency of exercise: none or irregular (1 or 2 episodes per week) and regular (more than 3 episodes per week).

Venous blood samples were collected after an overnight fast of at least 8 h, during baseline surveillance and every two years. Serum concentrations of glucose, total cholesterol, triglyceride, high-density lipoprotein (HDL) cholesterol, low-density lipoprotein (LDL) cholesterol, creatinine, aspartate aminotransferase (AST), alanine aminotransferase (ALT) and GGT were measured enzymatically using a Chemistry Analyzer (Hitachi 7600, Tokyo, Japan by August 2002 and ADVIA 1650; Bayer Diagnostics, Tarrytown, NY from September 2002) at baseline survey. For participants whose examination date was before September 2002, a conversion formula was used to reduce errors caused by changes in the measurement instrument. Estimated serum GGT level by ADVIA 1650 was − 2.98 + (1.004 × serum GGT level by HITACHI 7600). The intra-assay and inter-assay coefficients of variance (CVs) for GGT were 3.2% and 3.3%, respectively.

### Definition of metabolic syndrome

The modified National Cholesterol Education Program Adult Treatment Panel III was used to define MetS [17]. MetS was defined by the presence of three or more of the following risk factors: (1) waist circumference of > 90 cm in men and > 85 cm in women, (2) high triglycerides ≥ 150 mg/dL, (3) low HDL cholesterol < 40 mg/dL for men and < 50 mg/dL for women, (4) elevated systolic blood pressure ≥ 130 mmHg or elevated diastolic blood pressure ≥ 85 mmHg, and (5) high fasting plasma glucose ≥ 100 mg/dL. Individuals who reported taking anti-hypertensive, anti-diabetes, and/or triglyceride-lowering medications were considered to have elevated blood pressure, high fasting plasma glucose, and high triglycerides.

### Statistical analysis

Participants were divided according to quartiles of serum GGT levels in each sex as follows: Q1: ≤ 17, Q2: 18–27, Q3: 28–47, and Q4: ≥ 48 U/L for men and Q1: ≤ 9, Q2: 10–12, Q3:13–16, and Q4: ≥ 17 U/L for women. According to the GGT quartiles, the baseline characteristics of the study population were compared using Chi-squared tests for categorical variables and analysis of variance for continuous variables or Kruskal–Wallis tests depending on the normality of the distributions. Normal distribution was evaluated with determination of skewness using a Kolmogorov–Smirnov test. Serum triglyceride, AST, ALT, GGT, and creatinine levels and daily alcohol intake had skewed distributions, so these variables were expressed as median with interquartile range (IQR) in descriptive analysis and log-transformed prior to multiple Cox proportional hazards regression analysis. The Chi-square test was used to compare categorical variables and categorical data is shown as the frequency (%). Continuous data are presented as the mean (standard deviation [SD]) or median (IQR). The Log-rank test was used to show the Kaplan–Meier curves of cumulative MetS incidence according to sex-specific serum GGT quartiles. After setting the lowest quartile as the referent GGT group, multivariate Cox proportional hazards regression models were used to calculate hazard ratios (HRs) and 95% confidence intervals (CIs) for incident MetS adjusting for potential confounding variables. We have included the confounding variables in the logistic regression analysis model considering the commonly performed statistical principles to include established risk factors and statistically or marginally significant variables in the simple analysis. All analyses were performed using SAS statistical software (version 9.4; SAS Institute Inc., Cary, NC, USA). All P-values were two-tailed and statistical significance was set at P < 0.05.

## Results

Baseline characteristics of the total 5,960 study population according to GGT quartiles in each sex are described in Table 1. Waist circumference, systolic blood pressure, diastolic blood pressure, fasting plasma glucose, triglyceride, total cholesterol, and LDL cholesterol increased, whereas HDL-cholesterol increased with increasing serum GGT quartiles in both sexes. Moreover, current smoking, regular drinking, and antihypertensive medication were more frequently observed in the higher serum GGT quartile groups and serum AST and ALT were also elevated in the population with higher GGT quartiles.

**Table 1:** Baseline characteristics of study population by GGT quartiles in men and women

	GGT quartiles in men					GGT quartiles in women				
	Q1 (≤ 17)	Q2 (18–27)	Q3 (28–47)	Q4 (≥ 48)	P-value	Q1 (≤ 9)	Q2 (10–12)	Q3 (13–16)	Q4(≥ 17)	P-value
n	803	765	797	765		752	775	622	681	
Age (years)	53.1 (9.3)	51.4 (8.5)	50.5 (8.6)	51.2 (8.9)	< 0.001	48.4 (8.0)	50.3 (8.7)	50.7 (8.5)	51.5 (8.6)	< 0.001
Waist circumference (cm)	78.9 (6.8)	80.9 (6.5)	82.8 (6.4)	83.2 (6.0)	< 0.001	76.5 (7.8)	76.7 (8.1)	78.3 (8.9)	79.7 (8.7)	< 0.001
Systolic BP (mmHg)	116.7 (15.8)	117.2 (15.9)	120.3 (15.7)	121.1 (16.3)	< 0.001	111.7 (14.5)	113.4 (16.0)	113.4 (15.6)	117.2 (18.2)	< 0.001
Diastolic BP (mmHg)	77.9 (10.0)	78.6 (10.0)	81.0 (10.5)	81.8 (10.2)	< 0.001	73.3 (9.4)	74.6 (10.2)	75.0 (9.9)	76.9 (10.7)	< 0.001
Fasting plasma glucose (mg/dL)	83.7 (13.9)	85.7 (16.1)	86.4 (15.9)	89.2 (18.8)	< 0.001	79.0 (9.3)	80.8 (8.2)	82.4 (12.1)	83.0 (13.6)	< 0.001
Total cholesterol (mg/dL)	180.5 (31.2)	189.4 (32.6)	193.1 (39.0)	194.9 (34.7)	< 0.001	172.6 (28.8)	185.4 (33.4)	189.9 (32.9)	197.4 (33.8)	< 0.001
Triglyceride (mg/dL)	110 (83–139)	118 (94–148)	135 (105–176)	166 (122–237)	< 0.001	99 (82–124)	102 (82–127)	103 (82–133)	117 (95–144)	< 0.001
HDL-cholesterol (mg/dL)	47.7 (10.5)	45.6 (9.4)	44.7 (9.8)	44.5 (9.4)	< 0.001	50.0 (10.9)	49.0 (10.1)	48.8 (10.0)	47.9 (9.1)	0.001
LDL-cholesterol (mg/dL)	105.8 (38.9)	118.6 (31.1)	118.2 (34.1)	112.7 (28.5)	< 0.001	103.6 (25.6)	114.7 (30.3)	117.8 (30.7)	121.9 (30.5)	< 0.001
Creatinine (mg/dl)	0.93 (0.16)	0.95 (0.18)	0.95 (0.17)	0.93 1.17)	0.001	0.72 (0.08)	0.74 (0.11)	0.75 (0.11)	0.74 (0.11)	< 0.001
AST (U/L)	26 (23–30)	26 (23–30)	28 (25–33)	34 (29–43)	< 0.001	23 (21–26)	24 (21–27)	25 (1–28)	27 (23–32)	< 0.001
ALT (U/L)	21 (17–25)	24 (19–29)	27 (21–34)	37 (28–48)	< 0.001	16 (14–19)	17 (15–20)	19 (15–24)	24 (18–31)	< 0.001
Current smoker (%)	40.2	46.9	51.9	61.5	< 0.001	2.7	2.4	2.8	5.7	< 0.001
Regular drinker (%)^a^	51.7	65.8	80.9	88.2	0.001	25.4	27.8	31.6	36.6	< 0.001
Daily alcohol intake (g/day)	7.1 (2.6–16.8)	11.8 (3.5–26.1)	18.1 (6.9–34.7)	26.1 (12.4–47.2)	< 0.001	1.6 (0.7–3.5)	1.9 (0.7–4.4)	2.1 (0.9–4.1)	2.9 (1.0–8.1)	< 0.001
Regular exercise (%)^b^	29.1	28.6	27.0	26.5	0.616	26.2	23.8	23.4	21.7	0.472
Family history of diabetes (%)	7.6	11.5	10.7	10.7	0.050	11.4	11.0	11.7	16.0	0.010
Antihypertensive medication (%)	9.1	16.1	13.4	16.5	0.038	3.2	9.5	8.8	14.7	< 0.001
Antidiabetic medication (%)	8.1	5.9	3.8	6.3	0.205	1.3	1.9	2.9	3.6	0.041
Antilipidemic medication (%)	4.7	5.1	4.7	5.1	0.924	1.1	1.3	1.8	1.6	0.337

Data are expressed as the mean (SD), median (IQR) or percentage

BP, blood pressure; HDL, high density lipoprotein; hsCRP, high sensitivity C-reactive protein

P-values were calculated using ANOVA-test or chi-square test

^a^Regular drinker ≥ once/month

^b^Moderate intensity physical exercise ≥ three times/week

The incidence of MetS during 12 years of follow-up is presented in Table 2. During the follow-up period, the incidence rate of MetS was calculated biennially. During the 12-year follow-up period, a total of 2478 subjects (41.6%) developed MetS with an incidence rate every two years ranging from 5.0 to 12.4.

**Table 2:** Incidence of metabolic syndrome during the follow-up study

Year range	Follow-up	No.	Incidence cases (n)	Incidence rate per 2 years
2001–2002	Baseline	5960		
2003–2004	2 years	5624	545	9.7
2005–2006	4 years	4986	621	12.4
2007–2008	6 years	4423	407	9.2
2009–2010	8 years	4438	445	10.0
2011–2012	10 years	4163	210	5.0
2013–2014	12 years	3973	250	6.3

**Fig. 2:**
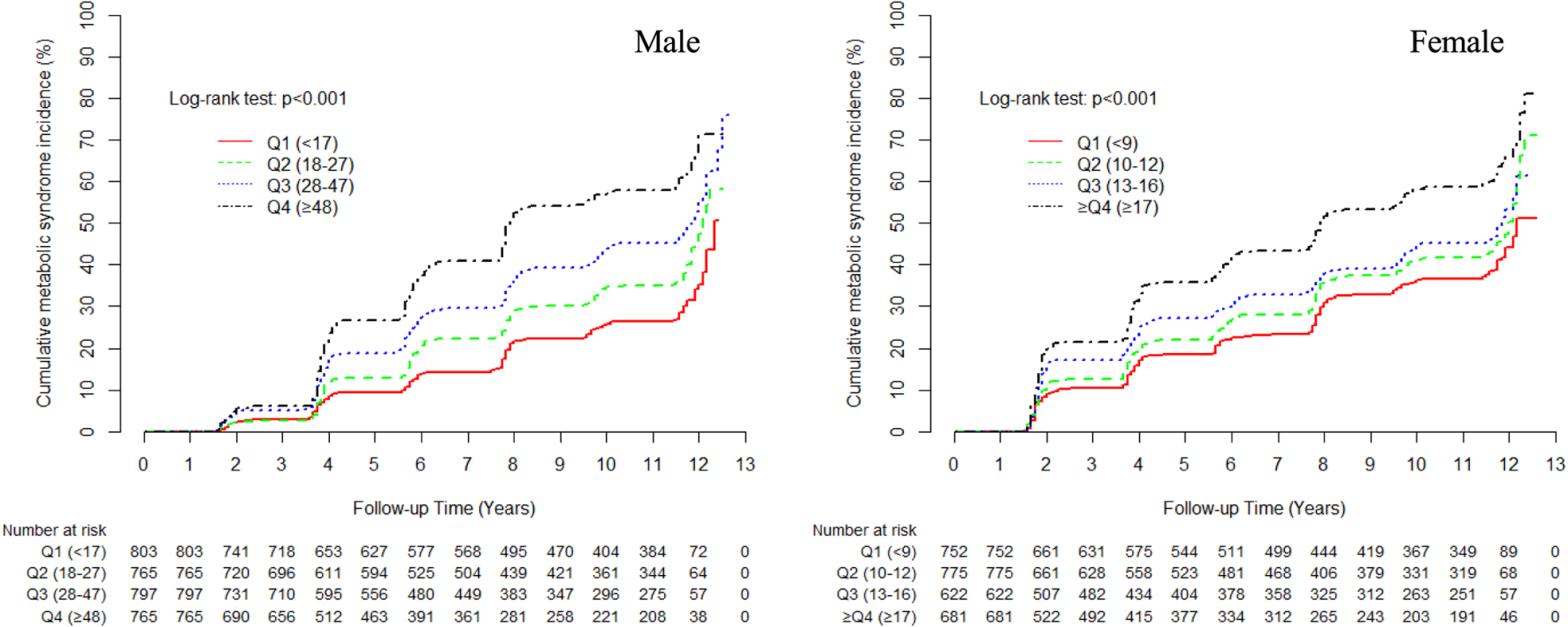
Cumulative incidence of MetS according to sex-specific quartiles of GGT levels in men and in women

The cumulative incidence of MetS according to serum GGT quartile is shown in Fig. 2 as a Kaplan–Meier curve. Higher GGT quartiles had a statistically higher cumulative incidence of MetS over 12 years in both men and women (All P values < 0.001). Table 3 shows the results of multivariate Cox proportional hazards regression analysis for the prediction of MetS by GGT quartiles. The incidence rate per 1000 person-years according to GGT quartiles increased proportionally with increasing serum GGT quartiles in both men and women. The HRs (95% CIs) for incident MetS for the highest quartile versus referent lowest quartile for serum GGT levels were 3.01 (2.35–3.76) for men and 1.83 (1.30–2.57) for women after adjusting for age, smoking status, daily alcohol intake (g/day), regular exercise, family history of diabetes, and log-transformed LDL-cholesterol, creatinine, and aminotransferase levels.

**Table 3:** Hazard ratios and 95% confidence intervals for incident metabolic syndrome by GGT quartiles in men and women

	GGT quartiles in men				GGT quartiles in women			
	Q1 (≤ 17)	Q2 (18–27)	Q3 (28–47)	Q4 (≥ 48)	Q1 (≤ 9)	Q2 (10–12)	Q3 (13–16)	Q4(≥ 17)
Total, n	803	762	797	765	752	775	622	681
New cases of type 2 diabetes, n	208	270	334	403	279	319	280	385
Mean follow-up (years)	8.7 (3.6)	8.5 (3.6)	7.7 (3.6)	6.9 (3.6)	8.3 (3.9)	7.8 (4.0)	7.6 (4.1)	6.6 (4.0)
Person-years of follow-up	6989	6513	6143	5298	6265	6054	4758	4488
Incidence rate per 1000 person-year	29.8	41.5	54.4	76.1	44.5	52.7	58.8	85.8
Model 1	1.00 (ref)	1.22 (0.94–1.58)	2.01 (1.59–2.54)	3.05 (2.33–3.73)	1.00 (ref)	1.15 (0.81–1.63)	1.17 (0.83–1.65)	1.76 (1.27–2.43)
Model 2	1.00 (ref)	1.21 (0.94–1.58)	2.00 (1.58–2.54)	3.04 (2.32–3.71)	1.00 (ref)	1.13 (0.79–1.60)	1.17 (0.83–1.64)	1.72 (1.24–2.39)
Model 3	1.00 (ref)	1.21 (0.94–1.58)	2.03 (1.60–2.58)	3.01 (2.35–3.76)	1.00 (ref)	1.10 (0.77–1.58)	1.11 (0.78–1.57)	1.83 (1.30–2.57)

Model 1: adjusted for age, smoking status, daily alcohol intake (g/day), and regular exercise

Model 2: adjusted for age, smoking status, daily alcohol intake (g/day), regular exercise, and family history of diabetes

Model 3: adjusted for age, smoking status, daily alcohol intake (g/day), regular exercise, family history of diabetes, and log-transformed LDL-cholesterol, creatinine, and aminotransferase levels

## Discussion

In this large, community-based longitudinal cohort study over 12 years, baseline serum GGT levels were independently positively related to incidence risk of MetS in both men and women after adjusting for potential confounding variables. Moreover, compared with women, the positive and dose–response relationships were more evidently observed in men. The positive association between GGT levels and MetS risk is compatible with the findings of previous studies. In a 2012 meta-analysis on serum GGT levels and incidence risk of MetS, Liu et al. [12] revealed a longitudinal relationship of serum GGT levels with MetS from nine prospective cohort studies. However, in their study, sex differences in GGT levels and MetS risk were not fully considered because most of included studies did not present results through sex-specific analyses. In a subgroup analyses from two prospective studies, the GGT levels was not significantly associated with MetS risk in women (RR 1.18, 95% CI 0.92–1.49; P = 0.187) [12]. Moreover, compared to the present study, most previous studies have relatively small numbers of participants at less than 1000 participants [18–20] or short follow-up periods within seven years [21, 22]. A prospective cohort study in the Unites States with long duration follow-up of 20 years among 3451 participants showed similar results to our study [23], but sex was not fully considered by not presenting separate data on both sexes. Considering the sex differences in GGT levels, sex-specification analyses models would more appropriate rather than merely adjusting for sex in the statistical analyses.

Previous studies among Korean adults also showed the positive associations between serum GGT levels and MetS. In a study of 211,725 Korean participants from the combined KoGES cohorts, Lee et al. [24]**,** showed the prevalence of MetS increased with increasing GGT quartiles in both men and women. However, the study was based on cross-sectional design and a temporal causality could not be established. In another study using the KoGES cohort among 2579 Korean adults, Yadav et al. [25], examined prospective associations between GGT levels and incident MetS and found the increment of GGT level as well as higher GGT level were positively associated with MetS risk. However, the study also had limitations, including the lack of consideration for previous liver disease at the baseline, a relatively small sample size (n = 2579), and short duration of follow-up (mean follow-up of 2.6 years). In this regard, our study confirmed that the longitudinal associations between serum GGT levels and MetS risk could be applied to both men and women through sex-specific multiple Cox proportional hazards regression analysis. Moreover, the inclusion of 5960 participants with a 12-year follow-up period in our study is a key strength of the present study.

Although many epidemiological studies have established that serum GGT levels are closely related to incident risk of MetS, how serum GGT is linked to the development of MetS remains unclear. However, the most conceivable hypothesis for the pathophysiology of the relationship between GGT levels and incident MetS risk is low-grade inflammation and insulin resistance [26]. Experimental and observational studies showed that GGT is associated with oxidative stress, low-grade inflammation, and insulin resistance, which are also closely linked to the development of MetS [5, 26]. Since GGT is a protective enzyme contributing to glutathione homeostasis, higher GGT levels may be a reflection of increased oxidative stress and chronic low-grade inflammation that is accompanied by the oxidative stress cascade from free radicals [9].

In addition, accumulating evidence suggests that oxidative stress is involved in the development of insulin resistance in animal models, which verifies the association between dysregulated glutathione metabolism and impaired insulin action in fat cells [27]. The alteration of inflammatory cytokines is crucial for the pathogenesis of type 2 diabetes [28], which is associated with a chronic low-grade inflammation state [29]. Moreover, genetic variation in GGT is likely a factor that increases type 2 diabetes risk. According to a Mendelian randomization study by Lee et al., GGT has an 11% higher risk of type 2 diabetes in a single instrumental variable analysis using the rs4820599 genetic variant of GGT1 in 7640 Koreans [30]. The suggested causal relationship between rs4820599 and type 2 diabetes was verified by single instrumental variable analysis in a two-sample Mendelian randomization analysis using the genetic relationship to type 2 diabetes from a trans-ethnic genome wide association study with 110,180 participants [30]. GGT could also be linked to MetS through hepatic steatosis. A previous study showed that elevation of serum GGT is associated with hepatic steatosis [31], which contributes to development of insulin resistance by altering the secretion of factors from the liver such as lipids and hepatokines including retinol-binding protein 4 and fetuin A and B [31–34]. This relationship between GGT and insulin resistance via hepatic steatosis could lead to the development of type 2 diabetes mellitus and MetS.

Another noteworthy finding in our study was that the HRs for MetS in accordance with GGT quartiles were higher in men than in women. In a previous study investigating the association with GGT and CVD, the effect size of GGT level on CVD risk was more prominent in men than in women [13, 14]. Although the reason for the discrepancy by sex in the longitudinal relationship of GGT levels with MetS remains unclear, some explanatory biological mechanisms may be offered. First, sex hormones have a significant impact on energy metabolism, body composition, vascular function, and inflammatory responses [35, 36]. Moreover, estradiol has antioxidant effects [6, 14]. Estrogen has various receptors in non-reproductive tissues throughout the human body and could decrease production of reactive oxygen species in mitochondria and pro-inflammatory cytokines such as tumor necrosis factor-α, which in turn reduces chronic low-grade inflammation [37, 38]. In addition to sex hormones, compared to women, men tend to have unfavorable lifestyle habits including higher rates of current smoking and drinking, which lead to sex differences in oxidative stress burden and counterregulatory elevation of GGT levels in men.

This study has several limitations that should be considered. First, the study population may not represent the general Korean population, as the participants were limited to specific geographic regions and age groups. Second, conditions that alternate serum GGT level including chronic heart failure [39], chronic obstructive pulmonary disease [40], chronic kidney disease [41], and medication history including antiepileptic drugs [42] were not fully taken into account in the study design. Third, we only utilized baseline levels of GGT and other liver enzymes and did not gather repeated measurements, which is a potential limitation because sequential changes in GGT levels could occur over time. Although, the prevalence of liver disease could vary during the follow-up period, the newly developed patients with viral or drug-induced hepatitis were not fully considered in the statistical analysis models [43, 44].

In conclusion, high levels of GGT were found to be associated with an increased risk of MetS in both men and women and these positive associations were stronger in men than in women.

## Data Availability

The dataset used in this study (Ansan-Ansung cohort) can be provided after review and evaluation of research plan by the Korea Centers for Disease Control and Prevention (http://www.cdc.go.kr/CDC/eng/main.jsp).
